# Assessment of *in vivo* versus *in vitro* biofilm formation of clinical methicillin-resistant *Staphylococcus aureus* isolates from endotracheal tubes

**DOI:** 10.1038/s41598-018-30494-7

**Published:** 2018-08-09

**Authors:** Laia Fernández-Barat, Soumaya Ben-Aicha, Anna Motos, Jordi Vila, Francesc Marco, Montserrat Rigol, Laura Muñoz, Gianluigi Li Bassi, Miquel Ferrer, Antoni Torres

**Affiliations:** 1 Centro de Investigación Biomedica En Red-Enfermedades Respiratorias (CibeRes, CB06/06/0028) and Institut d’Investigacions Biomèdiques August Pi i Sunyer (IDIBAPS), Barcelona, Spain; 2 School of Medicine, University of Barcelona, Barcelona, Spain; 3 Microbiology Service at Hospital Clinic and Institute of Global Health (ISGlobal) CRESIB Centre Esther Koplowitz, Barcelona, Spain; 4 Cardiology Service at Hospital Clinic, Barcelona, Spain; 5 Pulmonary and Critical Care Unit, Respiratory Institute, Hospital Clinic, Barcelona, Spain

## Abstract

Our aim was to demonstrate that biofilm formation in a clinical strain of methicillin-resistant *Staphylococcus aureus* (MRSA) can be enhanced by environment exposure in an endotracheal tube (ETT) and to determine how it is affected by systemic treatment and atmospheric conditions. Second, we aimed to assess biofilm production dynamics after extubation. We prospectively analyzed 70 ETT samples obtained from pigs randomized to be untreated (controls, n = 20), or treated with vancomycin (n = 32) or linezolid (n = 18). A clinical MRSA strain (MRSA-in) was inoculated in pigs to create a pneumonia model, before treating with antibiotics. Tracheally intubated pigs with MRSA severe pneumonia, were mechanically ventilated for 69 ± 16 hours. All MRSA isolates retrieved from ETTs (ETT-MRSA) were tested for their *in vitro* biofilm production by microtiter plate assay. *In vitro* biofilm production of MRSA isolates was sequentially studied over the next 8 days post-extubation to assess biofilm capability dynamics over time. All experiments were performed under ambient air (O_2_) or ambient air supplemented with 5% CO_2_. We collected 52 ETT-MRSA isolates (placebo N = 19, linezolid N = 11, and vancomycin N = 22) that were clonally identical to the MRSA-in. Among the ETT-MRSA isolates, biofilm production more than doubled after extubation in 40% and 50% under 5% CO_2_ and O_2_, respectively. Systemic antibiotic treatment during intubation did not affect this outcome. Under both atmospheric conditions, biofilm production for MRSA-in was at least doubled for 9 ETT-MRSA isolates, and assessment of these showed that biofilm production decreased progressively over a 4-day period after extubation. In conclusion, a weak biofilm producer MRSA strain significantly enhances its biofilm production within an ETT, but it is influenced by the ETT environment rather than by the systemic treatment used during intubation or by the atmospheric conditions used for bacterial growth.

## Introduction

Intubation with an endotracheal tube (ETT) is a routine procedure that is applied to 40%–50% of patients admitted to intensive care units (ICUs)^1^. However, when covered by secretions or cell debris, microorganisms from the stomach or oropharynx^2^ can rapidly colonize the ETT by directly or indirectly adhering to its surface^3,4^. The attachment of these microorganisms to the ETT surface changes gene expression to allow the bacteria to grow in a sessile mode that promotes biofilm formation^5^. Several studies have found a relationship between ETT biofilms and ventilator-associated pneumonia (VAP)^6–10^, but it is difficult to assess whether ETT colonization results directly from VAP or from concomitant microorganisms in the patients’ airways^11^. In any case, the ETT biofilm becomes a source of unnecessary pathogens in the critically ill patient.

*Staphylococcus aureus* is one of the most frequently isolated microorganisms in VAP^12^, and is especially challenging to treat in its oxacillin-resistant form (methicillin-resistant *S*. *aureus*, MRSA). Though decreasing overall, the prevalence of MRSA remains above 25% in many southern and eastern European countries. Therefore, it remains an important public health priority. A major concern of hospital-acquired MRSA infections is its ability to form biofilms on indwelling devices, which in turn, can result in invasive infection, sepsis, morbidity and mortality^13^. The ability to form a biofilm is considered a virulence factor since the production of surface poly-N-acetylglucosamine is associated with initial stages of colonization and contributes to immune system evasion^14^, even though microorganisms do not routinely undergo testing for this ability in clinical settings. Unfortunately, however, there are few clinically validated methods for testing biofilms^15–17^, although some exhibit interesting results^18^. The microtiter plate method^19–21^ is extensively used to quantify the *in vitro* biofilm capability of bacteria, but is limited by the inability to extrapolate those results to *in vivo* scenarios with confidence. A clinician may therefore reach erroneous conclusions when basing them exclusively on the *in vitro* method.

We designed our hypothesis based on previous studies demonstrating a thick layer of secretions and biofilm in ETT after several days of mechanical ventilation^3,4,6,22,23^, and based on the fact that many of the components found within an ETT after mechanical ventilation are well described environmental stress factors that activate the biofilm mode of growth^24^, such as: exposure to ETT surface, extracellular DNA, cells debris, mucus secretions, sublethal doses of antimicrobials, nutrients shortage, inflammatory response or impaired availability of O_2_ on some deep layers of respiratory secretions where biofilms are found. We hypothesized that strains with weak *in vitro* biofilm-producing abilities could have increased ability under *in vivo* settings. Therefore, based on the assumption that biofilm formation is a reversible trait, we focused on progressive biofilm production on an ETT several days after extubation for a clinical strain of MRSA (MRSA-in) that caused severe pneumonia in a model of tracheally intubated and mechanically ventilated pigs^25^. Additionally, experiments were performed under ambient air (O_2_) and under ambient air supplemented with 5% CO_2_ (5% CO_2_) to assess the influence of atmospheric conditions on biofilm formation. This reflected the clinical scenario where different proportion of gases can be applied during the mechanical ventilation of intubated patients^26^. Finally, because linezolid and vancomycin are the two recommended drugs for VAP caused by MRSA^27^, we assessed the effect of these two systemic treatments—when used during intubation—on the ability to form a biofilm after extubation. Although other factors may also play a role in ETT-biofilm formation we focused on three of them related with the clinical management of ICU patients such as: the endotracheal tube, the systemic antimicrobial treatment and atmospheric conditions that can vary between different ventilatory patterns applied to critical patients.

## Results

We assessed 35 ETTs (Mallinckrodt Hi-Lo; Mallinckrodt Medical, Athlone, Ireland) with 7.5 mm internal diameters from 10, 9 and 16 pigs treated with placebo (controls), linezolid and vancomycin, respectively. Fifty-two sessile MRSA isolates (placebo N = 19, linezolid N = 11, vancomycin N = 22) were obtained from these ETTs that were clonally identical to the inoculated MRSA (MRSA-in; data not shown).

**Figure 1:**
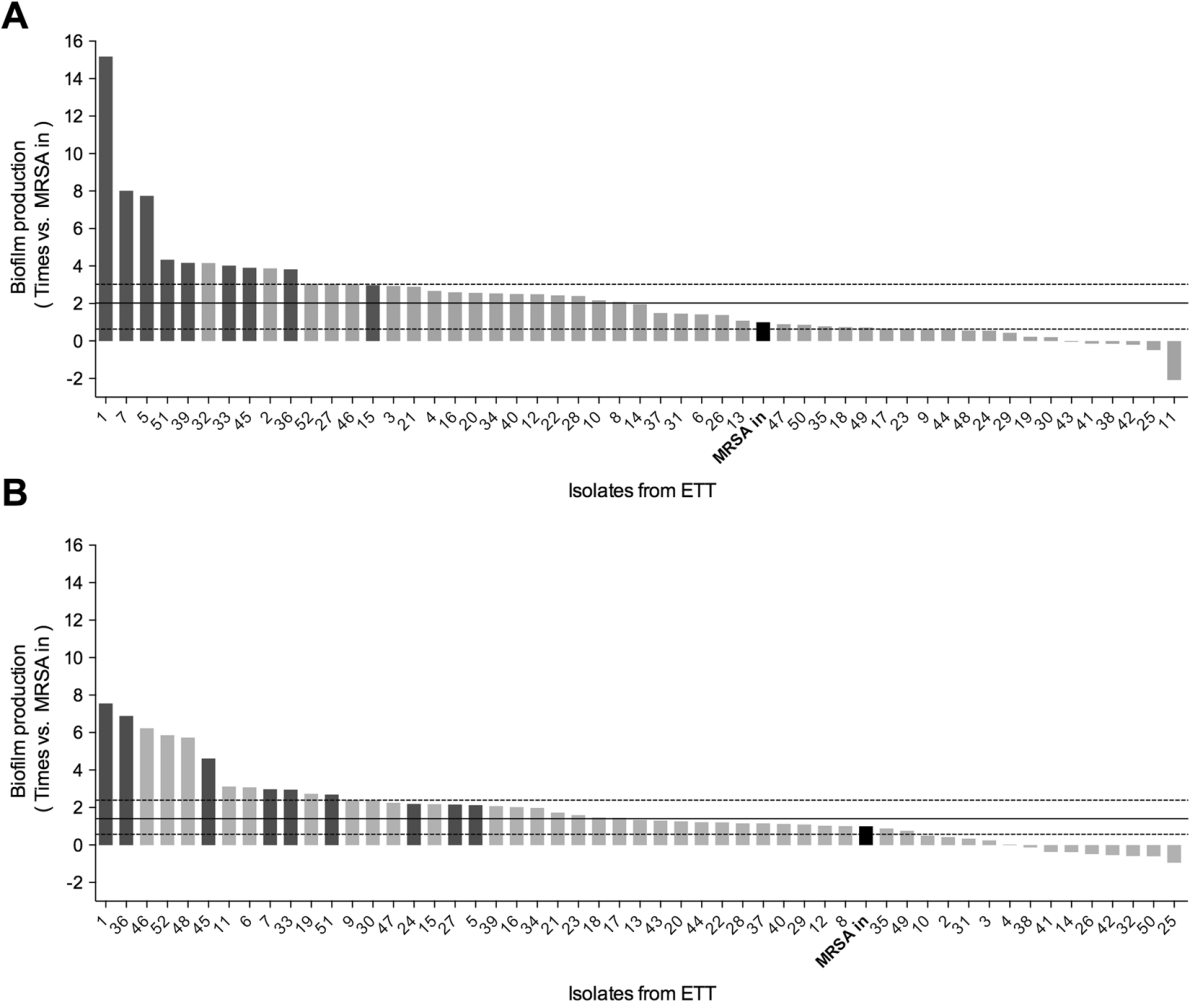
Biofilm production of 52 ETT-MRSA isolates compared with the MRSA-in under ambient air or ambient air with 5% CO_2._ Each bar represents the biofilm production of each ETT-MRSA isolate versus the MRSA-in (black bar). (**A**) Biofilm production O_2_ on day 2 after extubation (peak production); 50% of ETT-MRSA isolates increased biofilm production more than double that of MRSA-in. (**B**) Biofilm production under 5% CO_2_ on day 1 after extubation (peak production); 40% of ETT-MRSA isolates increased more than twice MRSA-in biofilm production. The highest biofilm producers ETT-MRSA isolates (n = 9), in dark gray, under both O_2_ and 5% CO_2_ were selected to undergo the biofilm production dynamics post-extubation. Abbreviations: 5% CO_2_, ambient air with 5% CO_2_; O_2_, ambient air; ETT-MRSA, clinical MRSA isolates from endotracheal tubes; MRSA-in, MRSA inoculated into pigs’ lungs; MRSA, methicillin-resistant *Staphylococcus aureus*.

**Figure 2:**
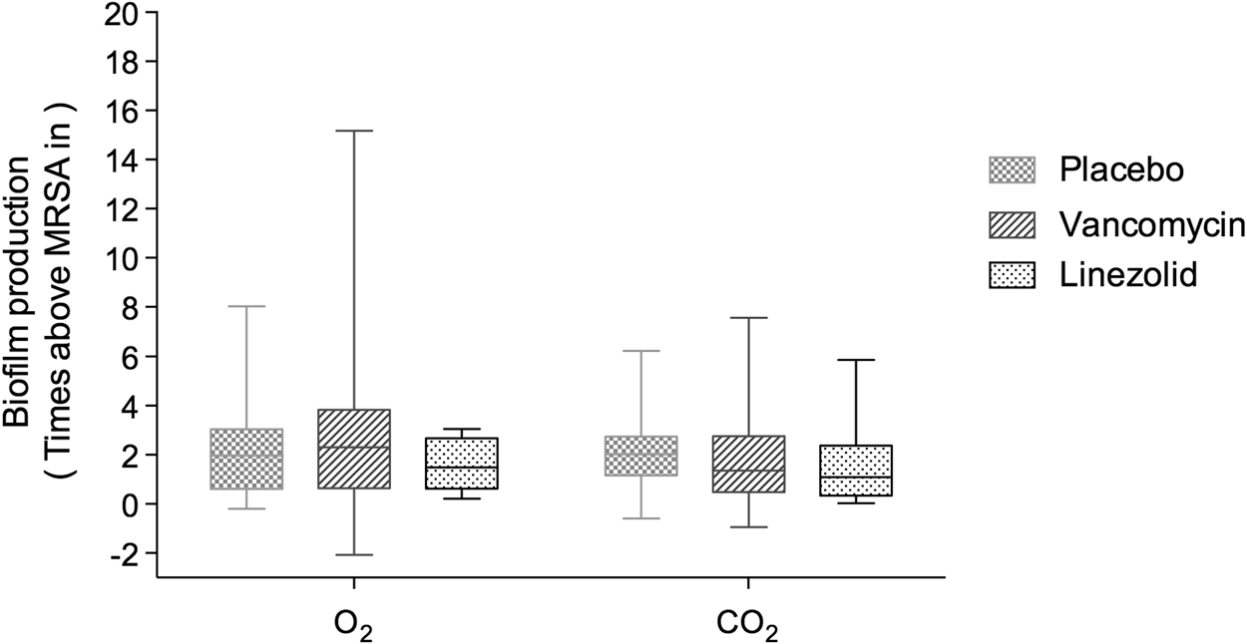
Effect of systemic antibiotic treatment on biofilm production in the 52 ETT-MRSA isolates under O_2_ or 5% CO_2_ conditions Median (interquartile range) values for biofilm production of the 52 ETT-MRSA compared with the MRSA-in under O_2_ (**A**) and 5% CO_2_ (**B**). Time of assessment: day of peak production. Biofilm production was not influenced by systemic treatment with placebo (n = 19), linezolid (n = 11), or vancomycin (n = 22) under either O_2_ (1.96 [0.61–3.02], 2.30 [0.64–3.83], and 1.49 [0.63–2.67], respectively; p = 0.92) or 5% CO_2_ (2.02 [1.16–2.34], 1.36 [0.48–2.75], and 1.09 [0.34–2.38], respectively; p = 0.62). Abbreviations: 5% CO_2_, ambient air with 5% CO_2_; O_2_, ambient air; ETT-MRSA, clinical MRSA isolates from endotracheal tubes; MRSA-in, MRSA inoculated into pigs’ lungs; MRSA, methicillin-resistant *Staphylococcus aureus*.

Overall, 61% and 71% of the MRSA isolates from within the ETTs showed a significant increase in biofilm production compared with the MRSA-in under O_2_ (2.02 [0.63–3.02], p < 0.001) and 5% CO_2_ (1.40 [0.57–2.39], p = 0.007), respectively (Fig. 1). However, biofilm production was not influenced by systemic treatment with linezolid, vancomycin, or placebo (Fig. 2). In O_2_ and 5% CO_2_ MRSA-in biofilm production doubled in 50% and 40% of ETT-MRSA isolates respectively. Of these, 9 MRSA isolates doubled biofilm production under both O_2_ and 5% CO_2_ conditions, and these were selected to assess progressive *in vitro* biofilm production for 8 days after extubation.

### Progressive biofilm production after extubation

**Figure 3:**
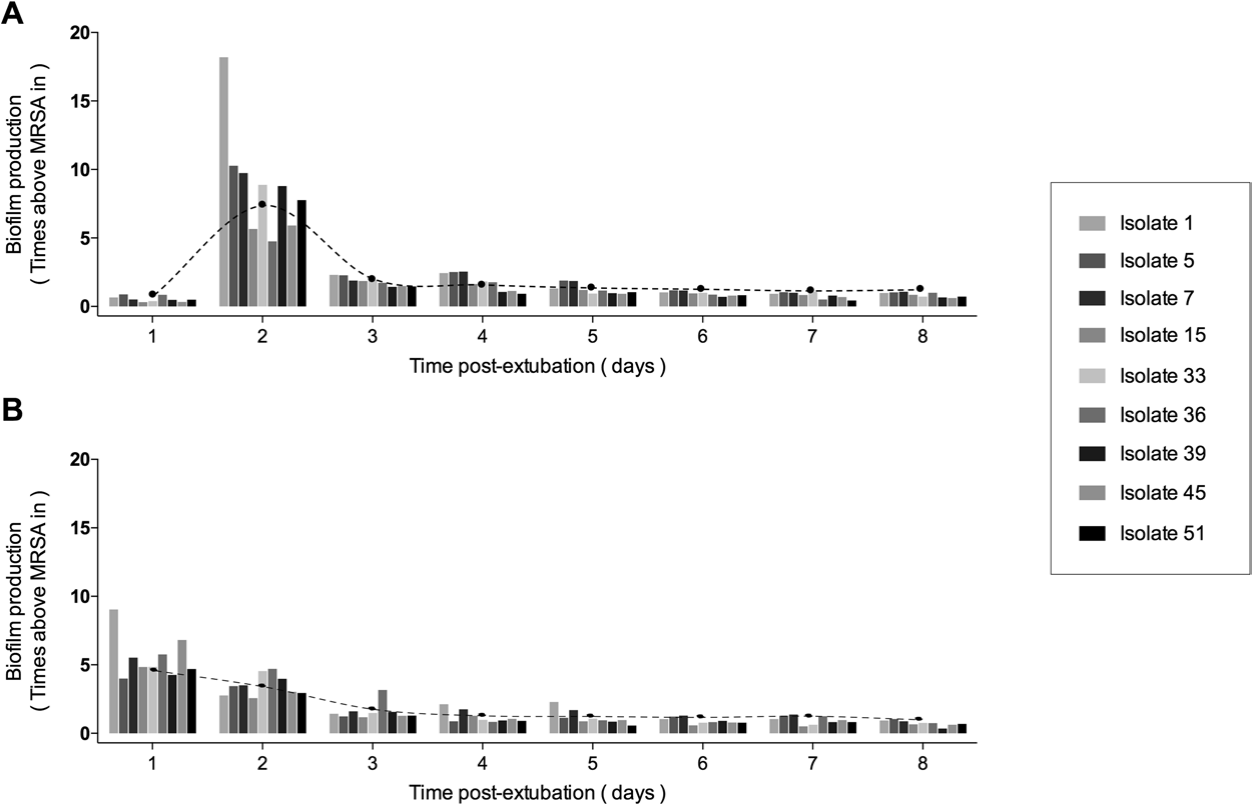
Biofilm production dynamics after extubation in 9 ETT-MRSA isolates under O_2_ or 5% CO_2_ Each color-bar represents the biofilm production of each ETT-MRSA isolate compared with the MRSA-in over days 1–8 after extubation. (**A**) Biofilm production dynamics under O_2_. Maximum biofilm production was on day 2. (**B**) Biofilm production dynamics under CO_2_. Maximum biofilm production was on day 1. Since the 5% CO_2_ atmosphere better mimics the atmospheric conditions of mechanical ventilation. When ETT-MRSA are rapidly switched from the ETT environment to O_2_ alone, they would need a day to adapt their metabolism to the new atmospheric conditions. Black points represent median biofilm production of the 9 isolates each day. *Abbreviations:* 5% CO_2_, ambient air with 5% CO_2_; O_2_, ambient air; ETT-MRSA, clinical MRSA isolates from endotracheal tubes; MRSA-in, MRSA inoculated into pigs’ lungs; MRSA, methicillin-resistant *Staphylococcus aureus*.

Biofilm production by the nine pre-selected ETT-MRSA isolates showed a significant and progressive decrease after extubation under both O_2_ and 5% CO_2_ conditions (Fig. 3). The peak biofilm production was under O_2_ at day 2 after extubation and was 7.78 times (6.62–9.67 times) that of the MRSA-in. By contrast, biofilm production under 5% CO_2_ peaked at day 1 after extubation and was only 4.46 times (3.21–7.71 times) that of the MRSA-in. Then, by stratifying time of assessment into ≤ 4 days and ≥ 4 days after extubation, we showed that a time lapse of 4 days was necessary to downregulate biofilm production to a baseline level.

**Figure 4:**
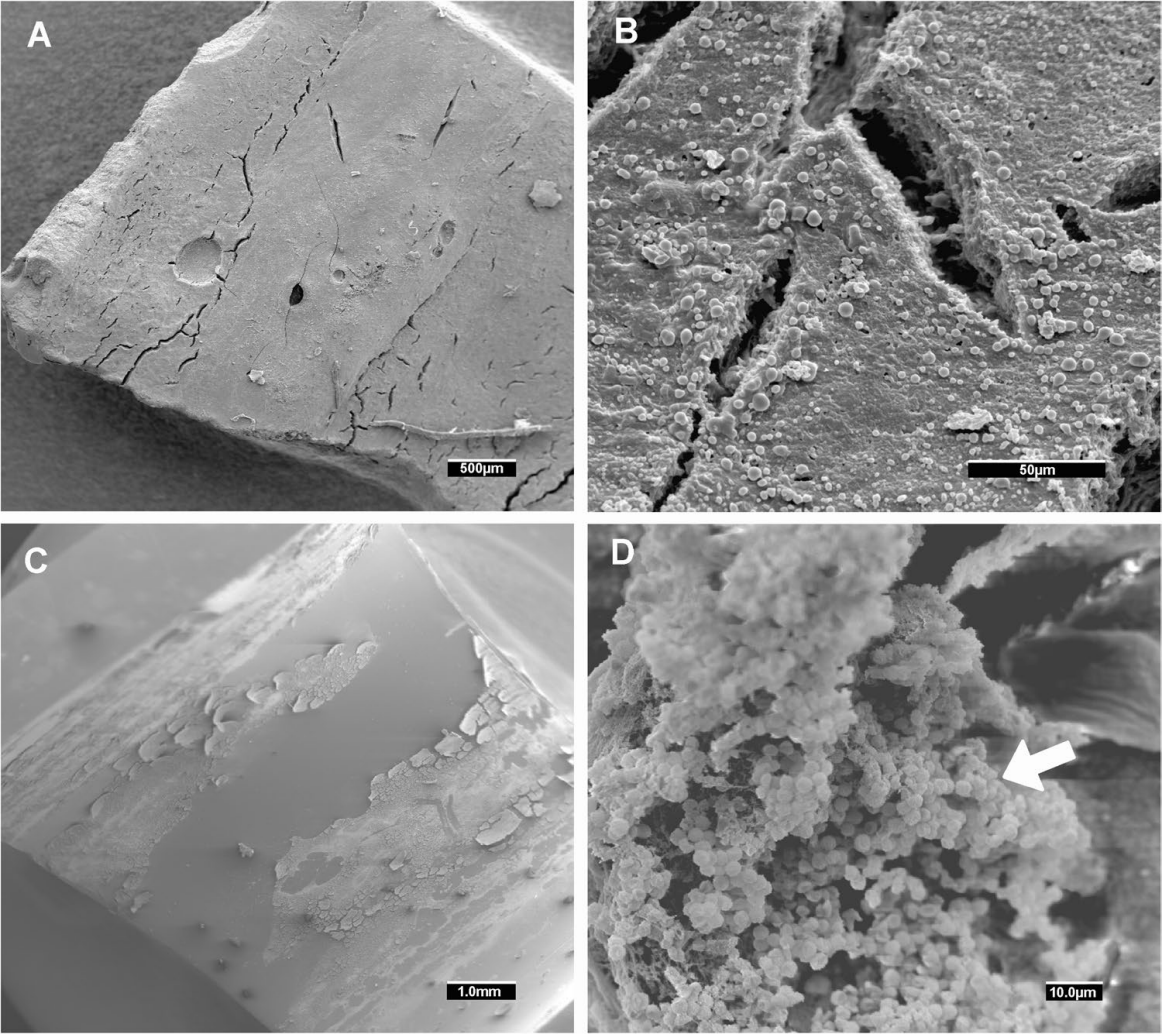
Representative scanning electron microscopy of *in vivo* MRSA biofilm (**A**) Isolate 1 showing an *in vivo* detached biofilm at low magnification. Sometimes the sample processing for scanning electron microscopy released the biofilm cluster from the endotracheal tube surface. At higher magnification (**B**), cocci morphologies can be distinguished. The pig from which we obtained Isolate 1 was treated with vancomycin. (**C**) Isolate 45 (from a placebo treated pig) showing an *in vivo* biofilm attached to the endotracheal tube at low magnification. (**D**) at higher magnification, a cocci biofilm cluster was found (white arrow). *Abbreviations:* MRSA, methicillin-resistant *Staphylococcus aureus*.

**Figure 5:**
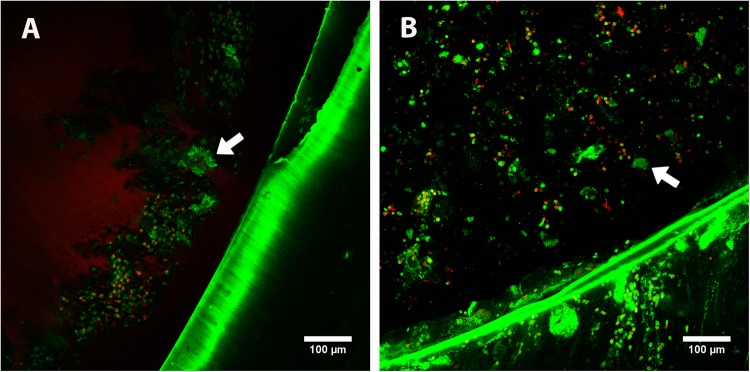
Representative confocal laser scanning microscopy of *in vivo* MRSA biofilm Biofilm clusters (white arrows) were stained with the LIVE/DEAD BacLight kit (INVITROGEN, Barcelona, Spain). Viable bacteria (stained green by SYTO 9) are visible, but dead bacteria (stained red by propidium iodide) were infrequently detected. The nuclei and cytoplasm of eukaryotic cells from the pig were also stained nonspecifically by propidium iodide and SYTO 9 (large red and green blotches, respectively). (**A**,**B**) correspond to the *in vivo* biofilms of Isolate 39 and Isolate 45 obtained from pigs treated with vancomycin and placebo, respectively. *Abbreviations:* MRSA, methicillin-resistant *Staphylococcus aureus*.

After day 4, biofilm production remained stable at a baseline level in both O_2_ (1.71 [0.88–3.53] ≤ 4 days vs 0.95 [0.76–1.10] ≥ 4 days, p < 0.001) and 5% CO_2_ (1.90 [1.08–3.77] ≤ 4 days vs 0.89 [0.68–1.08] ≥ 4 days, p < 0.001). Figures 4 and 5 show the *in vivo* ETT biofilms under scanning electron microscopy and confocal laser scanning microscopy for three representative ETTs.

### Biofilm production under O_2_ or 5% CO_2_

Our MRSA strains exhibited similar biofilm production dynamics over time under both O_2_ and 5% CO_2_ after extubation. However, peak biofilm production under O_2_ occurred 2 days after extubation, whereas peak biofilm production under CO_2_ occurred 1 day after extubation. Our strains exhibited increased biofilm production under O_2_ compared with 5% CO_2_, except on day 1, when biofilm production was higher under 5% CO_2_ than under O_2_. No differences were found in biofilm growth under O_2_ and 5% CO_2_ conditions between days 3, 6, and 7_._

## Discussion

We demonstrated that endotracheal intubation significantly increased the ability of an MRSA strain to form biofilms in a way not influenced by the systemic treatment received during intubation. This finding is of clinical relevance because a weak *in vitro* biofilm producer can become a strong *in vivo* biofilm producer. It was notable that this ability reverted significantly to a baseline level after several days of culture-plate passes, suggesting that the microenvironment within an ETT enhances biofilm formation. Thus, *in vitro* biofilm phenotype on microtiter plates does not mandatorily speak for the *in vivo* biofilm phenotype of a particular strain. In addition, we demonstrated how incubation with O_2_ rather than 5% CO_2_ can amplify biofilm formation of MRSA, but without affecting the dynamics of biofilm formation after extubation. Finally, our findings impact on using *in vitro* tests for validating, as far as they don’t exactly mimic *in vivo* conditions and in this sense our work emphasizes the use of tissue based models^28^, animal models, or clinical studies for biofilm studies and anti-biofilm technology effectiveness testing for medical devices like ETTs^3,29–31^.

The dilemma between using *in vitro* and *in vivo* models is an important issue when assessing biofilm-associated infections^32^. For an ICU clinician, it might be relevant to know the behavior of a particular strain within an ETT because of the known association with VAP occurrence^7,8,33^, but this information is not usually available. Although *in vitro* studies benefit from high reproducibility given their extremely controlled conditions, they are unable to reproduce the very clinical factors that can affect outcomes. However, *in vitro* studies using synthetic mucus or gel-entrapped bacteria have shown promising results^34–36^.

ETT clinical strains behave accordingly to the ETT environment immediately after extubation and a snapshot of the microorganism’s response can be provided based on their *in vitro* behavior. We demonstrated that a weak biofilm producer *in vitro* could have a stronger biofilm forming ability within an ETT. This can be attributed to the synergistic effect of respiratory secretions, based on evidence that mucus enhances biofilm production^34,37,38^, enables colonization^39^, and disrupts the effect of antiseptics^40^. However, the reasons why some MRSA isolates maintained or decreased their biofilm production capability or how these differences in biofilm production affected clinical outcomes remains unclear. Although this needs further investigation, variability in mucus secretions, host immune system responses^41^, sublethal concentration of antimicrobials^42^, and the underlying clinical conditions per subject will almost certainly have influenced the ETT biofilm formed by each MRSA strain^43^. Additionally, not only different panel of chemokines and cytokines during *S*. *aureus* acute versus biofilm infection models have been previously reported^44^, but how the proinflammatory response activated by *S*. *aureus* can enhance indeed the progress to chronic infection^45^.

ETT colonization occurs rapidly after intubation^2^. Although the biofilm mode of growth requires important changes in gene expression^46,47^, the dynamic changes in expression over time have been poorly investigated for *S*. *aureus*^41^. In the current study, MRSA isolates needed an average of four days to downregulate their biofilm capability to a baseline status, reinforcing the high plasticity of the biofilm mode of growth. Similarly, Fux *et al*. demonstrated^48^ that *S*. *aureus* reached a steady state biofilm after 4 days in a dynamic flow system. Further studies are needed to elucidate the main genes involved in this process, which could identify new therapeutic targets.

The biofilm mode of growth can be activated by sub-inhibitory concentrations of antimicrobial drugs^49,50^. For instance, sublethal doses of vancomycin or linezolid have been shown to induce biofilm formation in *S*. *aureus* infection^42,51^. In line with these findings, we previously found that systemic vancomycin therapy was associated with increased biofilm thickness^22^ and bacterial spread (area) *in vivo*^4^, and attributed those results to sub-therapeutic doses of vancomycin achieved in secretions, leading to increased infection severity, mucus production, and ETT biofilm formation. The present results, however, indicate that previous systemic treatment does not affect MRSA biofilm production *in vitro* after extubation. Thus, these findings suggest that previous exposure to systemic linezolid or vancomycin treatment during intubation did not produce irreversible genetic rearrangements in our model, and therefore did not affect biofilm formation after extubation. New studies to elucidate the impact of sublethal antibiotic doses on biofilm formation *in vivo* would be of relevance.

Different ventilatory settings and oxygen concentrations are applied to ICU patients during mechanical ventilation^26^. In our experiments, MRSA biofilm growth after extubation was progressively downregulated over time under both ambient air (O_2_) and ambient air with 5% CO_2_ environments. Our results are consistent with those of other authors who used similar methods to show that *S*. *aureus* strains showed significantly lower biofilm production when grown in a CO_2_-rich environment compared with an ambient air environment^52^. Using a different methodology, Ursic *et al*. found that MRSA biofilm production increased when grown in CO_2_-rich environments^53^. Interestingly, small colony variants of *S*. *aureus* have been described to grow in a CO_2_-dependent manner^54^. The fact that we did not find such variants, coupled with the different methodologies, may explain why our MRSA isolates exhibited lower biofilm production under 5% CO_2_.

The effects of atmospheric gases availability on gene expression rearrangement or biofilm composition has been reported previously^55,56^. Our finding of differences in MRSA response under O_2_ and 5% CO_2_ conditions on day 1 after extubation can be attributed to the fact that bacteria need to rearrange their metabolism from the ETT to the *in vitro* ambient air atmosphere. This metabolic rearrangement seems not to have occurred under 5% CO_2_ conditions, because the normal *in vivo* partial pressure of CO_2_ (35–45 mmHg) closely resembles that found in the *in vitro* 5% CO_2_ atmosphere (38 mmHg)^26^. Further research may elucidate the role of anaerobiosis on ETT-MRSA isolates, which we did not include in the present work. Other authors have demonstrated that anaerobiosis can stimulate *ica*-specific mRNA expression of some *S*. *aureus* species^57^.

This work has several limitations that deserve to be mentioned. First, we did not assess biofilm formation directly on the ETT-strain, which were frozen and plated before biofilm analysis. However, we previously demonstrated how ETT-MRSA growth and biofilm was not affected by freezing^4,6,22,23^, and also we performed the same freezing and plating step for all isolates, thereby removing any potential bias. Second, our ETT-MRSA strains were obtained from mechanically ventilated pigs instead of from ICU patients. Nevertheless, the MRSA-in strain used in the animal model was obtained from a patient, and therefore the results from our experimental model are reproducible and can be useful for future studies of clinical isolates from ICU patients.

In conclusion, our work shows how the biofilm-producing ability of a MRSA strain is influenced by the ETT environment *in vivo* rather than by systemic antibiotic treatment during intubation or atmospheric conditions during bacterial growth. In addition, this biofilm upregulation is reversible: with longer exposure to *in vitro* conditions the ability to form a biofilm decreased, and eventually returned to the baseline level.

## Materials and Methods

### Subjects

Pigs received an intrabronchial challenge with 75 mL of 10^6^ colonies forming units (CFU)/mL of a pathogenic Panton–Valentine leukocidin negative clinical strain of MRSA (MRSA-in), agr II, and ST 125 type, susceptible to vancomycin and linezolid. Following clinical pneumonia diagnosis, animals were randomized to be treated with vancomycin (15 mg/kg every 12 hrs intravenously), linezolid (10 mg/kg every 12 hrs intravenously), or 0.9% saline (placebo/controls) and mechanically ventilated for 69 ± 16 hours, as previously reported^22,58^. The MRSA-in and the MRSA isolates retrieved from ETT microbiological cultures were stored at −80 °C until analysis.

### MRSA genotyping

The relatedness of all the MRSA isolates was assessed by pulsed-field gel electrophoresis (PFGE), following previously published methods^59,60^. Briefly, a washed bacterial suspension was mixed with 1.8% agarose (FMC BioProducts, USA) at 50 °C and allowed to solidify into plug molds for 10 min at 4 °C. Chromosomal DNA was prepared over several incubating and washing steps, using ESP (0.5 M EDTA, 1% sarkosyl), lysostaphin 400 µg/mL (SIGMA-ALDRICH, Spain), and proteinase K (ROCHE diagnostics S.L., Spain). DNA fragments generated by Sma I (SIGMA-ALDRICH, Spain) were separated by PFGE, using a CHEF-DRII apparatus (BIO-RAD, Richmond, CA, USA). The pulse times were increased linearly over 20 hrs from 5 s to 40 s at 200 V. The MRSA-in strain was used as a molecular size DNA marker, because the aim was to demonstrate that the ETT-MRSA isolates and the MRSA-in exhibited the same PFGE patterns. Following electrophoresis, the gel was stained with SYBR Safe (THERMO FISHER scientific, Spain) and photographed. To analyze the PFGE patterns, we used Bio-Rad software (Diversity Database, BIO-RAD, Richmond, CA, USA).

### Microtiter plate assay

The *in vitro* ability of all isolated MRSA strains to form biofilm was assessed by microtiter plate assay of Christensen G. slightly modified as follows^19^. The MRSA-in and all ETT-MRSA isolates were first cultured overnight at 37 °C with agitation on tryptic soy broth media (TSB; SIGMA-ALDRICH, Spain); After this, each isolate was diluted 1/50 in 200 μL TSB with 0.25% glucose in a micro well of a 96 flat bottom microtiter plate (polystyrene, sterile; SIGMA-ALDRICH, Spain) and incubated, without shaking, overnight at 37 °C. Then, the medium was removed, 200 μL of 0.1% safranin (SIGMA-ALDRICH, Spain) were added to stain the biofilm over 1 min. Then, the saturated dye and non-adherent bacteria were removed by rinsing with 200 μL phosphate-buffered saline, three times. The optical density of biofilm was measured in a Synergy 2 Multimode Microplate Reader (BIOTEK Instruments, Inc., USA) at a wavelength of 490 nm. For each MRSA isolate and condition tested we performed four independent experiments, with three intra-assay replicates each.

### Assessment of biofilm capability over time

MRSA isolates were first unfrozen and cultured on blood agar plates. Then, we plated up to eight different passes over 8 days after extubation to allow gene expression to adapt from the ETT to the culture plates. In so doing, we aimed to determine if their ability to form a biofilm was reversed to baseline level after several *in vitro* plate-cultures post-extubation. This was performed separately under O_2_ and 5% CO_2_ conditions. Although we assessed biofilm capability for all MRSA isolates over the first two days, we only included the higher biofilm producers for all 8 days.

### Microscopy image acquisition

To assess the biofilm clusters *in vivo*, we performed scanning electron microscopy (SEM) and confocal laser scanning microscopy (CLSM) as previously reported^4,22^. CLSM and SEM images were obtained with a Leica TCS SP5 laser scanning confocal system (LEICA Microsystems Heidelberg GmbH, Manheim, Germany) equipped with a DMI6000 inverted microscope and a scanning electron microscope DSM 940 A (ZEISS, Oberkochen, Germany), respectively.

### Statistical analyses

Data are reported as the median (interquartile range, IQR) or as mean ± SD, and were tested for normal distribution using the Shapiro–Wilk test. Qualitative or categorical variables were compared between groups with the Mann–Whitney test. Paired variables were assessed using the non-parametric Wilcoxon signed ranks test and independent samples were assessed using the non-parametric Kruskal–Wallis test. The Bonferroni correction was used for all post-hoc comparisons. To determine the relationship between quantitative variables, the Spearman rank-order correlation coefficient was used. A two-sided *p-value* < 0.05 was considered statistically significant. All statistical analyses were performed using IBM SPSS, Version 21 software (IBM SPSS statistics, 21, Chicago, IL, USA).

### Ethical approval

The institutional review board and animal ethics committee approved all included studies. The project license number that covered the animal experiments were the following: Ethical Board of animal experimentation of the University of Barcelona (code: 296/09) and Ethical Board of the Hospital Clinic of Barcelona (code: 2009/5409). Animals were managed according to the National Institutes of Health guidelines for the Use and Care of Animals^61^.

## References

[1] Esperatti M (2010). Nosocomial Pneumonia in the Intensive Care Unit Acquired during Mechanical Ventilation or Not. Am. J. Respir. Crit Care Med..

[2] Perkins SD, Woeltje KF, Angenent LT (2010). Endotracheal tube biofilm inoculation of oral flora and subsequent colonization of opportunistic pathogens. Int. J. Med. Microbiol..

[3] Fernandez-Barat L, Torres A (2016). Biofilms in ventilator-associated pneumonia. Future. Microbiol..

[4] Fernandez-Barat L (2012). Direct analysis of bacterial viability in endotracheal tube biofilm from a pig model of methicillin-resistant Staphylococcus aureus pneumonia following antimicrobial therapy. FEMS Immunol. Med. Microbiol..

[5] Costerton JW, Stewart PS, Greenberg EP (1999). Bacterial biofilms: a common cause of persistent infections. Science.

[6] Li BG (2015). Endotracheal tube biofilm translocation in the lateral Trendelenburg position. Crit Care.

[7] Gil-Perotin S (2012). Implications of endotracheal tube biofilm in ventilator-associated pneumonia response: a state of concept. Crit Care.

[8] Adair CG (1999). Implications of endotracheal tube biofilm for ventilator-associated pneumonia. Intensive Care Med.

[9] Feldman C (1999). The presence and sequence of endotracheal tube colonization in patients undergoing mechanical ventilation. Eur Respir J.

[10] Inglis TJ, Millar MR, Jones JG, Robinson DA (1989). Tracheal tube biofilm as a source of bacterial colonization of the lung. J Clin Microbiol.

[11] Torres A (1995). Re-intubation increases the risk of nosocomial pneumonia in patients needing mechanical ventilation. Am J Respir Crit Care Med.

[12] European Centre for Disease Prevention and Control. Annual Epidemiological Report. http://ecdc.europa.eu/en/publications/_layouts/forms/Publication_DispForm.aspx?Li st=4f55ad51-4aed-4d32-b960-af70113dbb90&ID=1292 (2014).

[13] Silva-Santana G, Lenzi-Almeida KC, Lopes VGS, guiar-Alves F (2016). Biofilm formation in catheter-related infections by Panton-Valentine leukocidin-producing Staphylococcus aureus. Int Microbiol..

[14] Kropec A (2005). Poly-N-acetylglucosamine production in Staphylococcus aureus is essential for virulence in murine models of systemic infection. Infect. Immun..

[15] Pantanella F, Valenti P, Natalizi T, Passeri D, Berlutti F (2013). Analytical techniques to study microbial biofilm on abiotic surfaces: pros and cons of the main techniques currently in use. Ann. Ig.

[16] Hassan A (2011). Evaluation of different detection methods of biofilm formation in the clinical isolates. Braz. J Infect. Dis..

[17] Peeters E, Nelis HJ, Coenye T (2008). Comparison of multiple methods for quantification of microbial biofilms grown in microtiter plates. J Microbiol. Methods.

[18] Olivares E (2016). The BioFilm Ring Test: a Rapid Method for Routine Analysis of Pseudomonas aeruginosa Biofilm Formation Kinetics. J Clin. Microbiol..

[19] Christensen GD (1985). Adherence of coagulase-negative staphylococci to plastic tissue culture plates: a quantitative model for the adherence of staphylococci to medical devices. J Clin. Microbiol..

[20] Elkhatib WF, Khairalla AS, Ashour HM (2014). Evaluation of different microtiter plate-based methods for the quantitative assessment of Staphylococcus aureus biofilms. Future. Microbiol..

[21] Knobloch JK, Horstkotte MA, Rohde H, Mack D (2002). Evaluation of different detection methods of biofilm formation in Staphylococcus aureus. Med Microbiol. Immunol..

[22] Fernandez-Barat L (2012). Linezolid limits burden of methicillin-resistant Staphylococcus aureus in biofilm of tracheal tubes. Crit Care Med..

[23] Aguilera XE (2015). Tracheal tube biofilm removal through a novel closed-suctioning system: an experimental study. Br. J. Anaesth..

[24] Bui LM, Turnidge JD, Kidd SP (2015). The induction of Staphylococcus aureus biofilm formation or Small Colony Variants is a strain-specific response to host-generated chemical stresses. Microbes. Infect..

[25] Martinez-Olondris P (2010). An experimental model of pneumonia induced by methicillin-resistent Staphylococcus aureus in ventilated piglets. Eur Respir J.

[26] Tobin,M.J. *Principles and practice of mechanical ventilation* (The McGraw-Hill Companies, Inc., New York, 2013).

[27] American Thoracic Society & Infectious Diseases Society of America Guidelines for the Management of Adults with Hospital-acquired, Ventilator-associated, and Healthcare-associated Pneumonia. *Am J Respir Crit Care Med* **171**, 388–416 (2005). 10.1164/rccm.200405-644ST 15699079

[28] Wang Y, Leng V, Patel V, Phillips KS (2017). Injections through skin colonized with Staphylococcus aureus biofilm introduce contamination despite standard antimicrobial preparation procedures. Sci. Rep..

[29] Bardes JM, Gray D, Wilson A (2017). Effect of the endOclear((R)) Device on Biofilm in Endotracheal Tubes. Surg. Infect. (Larchmt.).

[30] Berra L (2012). A clinical assessment of the Mucus Shaver: a device to keep the endotracheal tube free from secretions. Crit Care Med..

[31] Pinciroli R (2016). Endotracheal Tubes Cleaned With a Novel Mechanism for Secretion Removal: A Randomized Controlled Clinical Study. Respir. Care.

[32] Lebeaux D, Chauhan A, Rendueles O, Beloin C (2013). From *in vitro* to *in vivo* Models of Bacterial Biofilm-Related Infections. Pathogens..

[33] Danin PE (2015). Description and microbiology of endotracheal tube biofilm in mechanically ventilated subjects. Respir. Care.

[34] Haley CL, Colmer-Hamood JA, Hamood AN (2012). Characterization of biofilm-like structures formed by Pseudomonas aeruginosa in a synthetic mucus medium. BMC. Microbiol..

[35] Sriramulu DD, Lunsdorf H, Lam JS, Romling U (2005). Microcolony formation: a novel biofilm model of Pseudomonas aeruginosa for the cystic fibrosis lung. J Med Microbiol..

[36] Pabst B, Pitts B, Lauchnor E, Stewart PS (2016). Gel-Entrapped Staphylococcus aureus Bacteria as Models of Biofilm Infection Exhibit Growth in Dense Aggregates, Oxygen Limitation, Antibiotic Tolerance, and Heterogeneous Gene Expression. Antimicrob. Agents Chemother..

[37] Landry RM, An D, Hupp JT, Singh PK, Parsek MR (2006). Mucin-Pseudomonas aeruginosa interactions promote biofilm formation and antibiotic resistance. Mol. Microbiol..

[38] Worlitzsch D (2002). Effects of reduced mucus oxygen concentration in airway Pseudomonas infections of cystic fibrosis patients. J. Clin. Invest.

[39] Gries DM, Pultz NJ, Donskey CJ (2005). Growth in cecal mucus facilitates colonization of the mouse intestinal tract by methicillin-resistant Staphylococcus aureus. J Infect. Dis..

[40] Ansorg RA, Azem T, Fabry WH, Rath PM (2002). Influence of mucin on the activity of the antiseptic Lavasept against Staphylococcus aureus. Chemotherapy.

[41] Scherr TD (2013). Global transcriptome analysis of Staphylococcus aureus biofilms in response to innate immune cells. Infect. Immun..

[42] Hsu CY (2011). Vancomycin promotes the bacterial autolysis, release of extracellular DNA, and biofilm formation in vancomycin-non-susceptible Staphylococcus aureus. FEMS Immunol. Med Microbiol..

[43] Jaffe A, Bush A (2001). Anti-inflammatory effects of macrolides in lung disease. Pediatr. Pulmonol..

[44] Brady RA, Mocca CP, Plaut RD, Takeda K, Burns DL (2018). Comparison of the immune response during acute and chronic Staphylococcus aureus infection. PLoS. One..

[45] Prabhakara R (2011). Suppression of the inflammatory immune response prevents the development of chronic biofilm infection due to methicillin-resistant Staphylococcus aureus. Infect. Immun..

[46] Rohde H (2007). Polysaccharide intercellular adhesin or protein factors in biofilm accumulation of Staphylococcus epidermidis and Staphylococcus aureus isolated from prosthetic hip and knee joint infections. Biomaterials.

[47] Delaune A (2012). The WalKR system controls major staphylococcal virulence genes and is involved in triggering the host inflammatory response. Infect. Immun..

[48] Fux CA, Wilson S, Stoodley P (2004). Detachment characteristics and oxacillin resistance of Staphyloccocus aureus biofilm emboli in an *in vitro* catheter infection model. J. Bacteriol..

[49] Hoffman LR (2005). Aminoglycoside antibiotics induce bacterial biofilm formation. Nature.

[50] Mesak LR, Miao V, Davies J (2008). Effects of subinhibitory concentrations of antibiotics on SOS and DNA repair gene expression in Staphylococcus aureus. Antimicrob. Agents Chemother..

[51] Ferrer MD (2017). Effect of antibiotics on biofilm inhibition and induction measured by real-time cell analysis. J Appl. Microbiol..

[52] Stepanovic S, Djukic N, Djordjevic V, Djukic S (2003). Influence of the incubation atmosphere on the production of biofilm by staphylococci. Clin. Microbiol. Infect..

[53] Ursic V, Tomic V, Kosnik M (2008). Effect of different incubation atmospheres on the production of biofilm in methicillin-resistant Staphylococcus aureus (MRSA) grown in nutrient-limited medium. Curr. Microbiol..

[54] Gomez-Gonzalez C (2010). Clinical and molecular characteristics of infections with CO2-dependent small-colony variants of Staphylococcus aureus. J Clin. Microbiol..

[55] Fernandez-Barat L (2017). Phenotypic shift in Pseudomonas aeruginosa populations from cystic fibrosis lungs after 2-week antipseudomonal treatment. J Cyst. Fibros..

[56] Asai K (2015). Effect of incubation atmosphere on the production and composition of staphylococcal biofilms. J Infect. Chemother..

[57] Cramton SE, Ulrich M, Gotz F, Doring G (2001). Anaerobic conditions induce expression of polysaccharide intercellular adhesin in Staphylococcus aureus and Staphylococcus epidermidis. Infect. Immun..

[58] Martinez-Olondris, P. *et al*. Efficacy of linezolid compared to vancomycin in an experimental model of pneumonia induced by methicillin-resistant Staphylococcus aureus in ventilated pigs. *Crit Care Med* (*2*011). 10.1097/CCM.0b013e31822d74a2 21926613

[59] Sierra JM, Marco F, Ruiz J, Jimenez de Anta MT, Vila J (2002). Correlation between the activity of different fluoroquinolones and the presence of mechanisms of quinolone resistance in epidemiologically related and unrelated strains of methicillin-susceptible and -resistant Staphylococcus aureus. Clin. Microbiol. Infect..

[60] Gautom RK (1997). Rapid pulsed-field gel electrophoresis protocol for typing of Escherichia coli O157:H7 and other gram-negative organisms in 1 day. J Clin. Microbiol..

[61] National Research Council. Guide for the Care and Use of Laboratory Animals: Eight Edition. Washington, DC: The National Academies Press. 10.17226/12910 (2011).

